# From Therapy Resistance to Targeted Therapies in Prostate Cancer

**DOI:** 10.3389/fonc.2022.877379

**Published:** 2022-05-24

**Authors:** Filipa Moreira-Silva, Rui Henrique, Carmen Jerónimo

**Affiliations:** ^1^ Cancer Biology and Epigenetics Group, Research Center of IPO Porto (CI-IPOP)/RISE@CI-IPOP (He-alth Research Network), Portuguese Oncology Institute of Porto (IPO Porto)/Porto Comprehensive Cancer Centre (Porto.CCC), Porto, Portugal; ^2^ Department of Pathology, Portuguese Oncology Institute of Porto (IPO Porto), Porto, Portugal; ^3^ Department of Pathology and Molecular Immunology, School of Medicine and Biomedical Sciences of the University of Porto (ICBAS-UP), Porto, Portugal

**Keywords:** prostate cancer, castration-resistant prostate cancer, therapy resistance, targeted therapies, epigenetics

## Abstract

Prostate cancer (PCa) is the second most common malignancy among men worldwide. Although early-stage disease is curable, advanced stage PCa is mostly incurable and eventually becomes resistant to standard therapeutic options. Different genetic and epigenetic alterations are associated with the development of therapy resistant PCa, with specific players being particularly involved in this process. Therefore, identification and targeting of these molecules with selective inhibitors might result in anti-tumoral effects. Herein, we describe the mechanisms underlying therapy resistance in PCa, focusing on the most relevant molecules, aiming to enlighten the current state of targeted therapies in PCa. We suggest that selective drug targeting, either alone or in combination with standard treatment options, might improve therapeutic sensitivity of resistant PCa. Moreover, an individualized analysis of tumor biology in each PCa patient might improve treatment selection and therapeutic response, enabling better disease management.

## Introduction

Currently, prostate cancer (PCa) constitutes the second most common malignancy and the fifth leading cause of cancer-related death in men, worldwide (1). PCa is a highly heterogeneous disease (2), characterized by several genetic and epigenetic alterations (2, 3), some of which can be used to assist treatment decision-making (3). Localized disease arises from luminal cells’ proliferation (2), being characterized by a slow growth and hormone-responsiveness, more common in elderly men (3). At the time of diagnosis, 80% of all the tumors are confined to the prostate gland (2) and roughly 50% harbor the well-known gene fusion *TMPRSS2:ERG* (3–5), implicated in PI3K signaling pathway aberrant activation (3, 6), *AR* overexpression, *PTEN* loss (6) and deregulation of epigenetic players’ encoding genes (3). Genetic alterations might also occur, specifically in *SPOP, TP53, ATM, MED12* and *FOXA1* genes (3). Furthermore, epigenetics also plays a role in prostate carcinogenesis, with DNA hypermethylation as one of the first alterations observed at low stages (7). Herein, one of the most well-known promotor’s hypermethylated gene is the *GSTP1*, which occurs in 90% of the tumors (8). Interestingly, this alteration is also observed in 50% of the PCa precursor lesions, suggesting this as an early event in prostate carcinogenesis (8). Additionally, histone deacetylases (HDACs) overexpression frequently detected in high-grade disease, particularly HDAC1 and HDAC2, has been associated with increased cell proliferation (9).

In locally advanced PCa, tumor cells invade the extra-prostatic tissue and/or metastasize to regional lymph nodes, paving the way to metastatic dissemination at distant organs, most commonly to the bones, liver, and lungs (2). Several genome-wide copy-number alterations have been observed, particularly *MYC* overexpression and *PTEN* and *SMAD4* deletion, which drives genomic instability and tumor progression (3). Specific epigenetic alterations similarly drive PCa progression, including EZH2 overexpression (2), *RASSF1A* promoter methylation (10) and overall hypomethylation (11).

Eventually, in due course of disease, PCa becomes resistant to androgen-deprivation therapy (ADT) – castration resistant PCa (CRPC) – disclosing raising serum PSA levels and/or clinical/imagiological tumor progression despite testosterone castrate levels (12). Interestingly, alterations in *AR*, *TP53*, *PTEN*, *RB1*, *ETS2*, DNA repair and chromatin and histone modifying genes are commonly found in CRPC (13–15). Moreover, it is observed amplification of the AR co-activator *NCOA2* and deletion of the AR co-repressor *LATS2* (13, 15). Furthermore, high DNA methylation levels (15), and overexpression of HDAC1, HDAC2, HDCA3, EZH2 (16), G9a (17) and LSD1 (18) have also been associated with CRPC.

Approximately 17% of tumors from CRPC patients eventually become AR indifferent (19, 20), progressing to a neuroendocrine PCa (NEPC) state, that does not respond to hormone therapy (19). NEPC harbors several genetic alterations, including *TMPRSS2:ERG* fusion, *MYC* and *AKT* overexpression, *PTEN* and *RB1* loss, and *TP53* mutations (2, 12, 21, 22). Moreover, epigenetic alterations, such as DNA hypermethylation as well as EZH2 and bromodomain and extra-terminal motif (BET) proteins overexpression have been found in NEPC (12).

### Standard of Care in Prostate Cancer Treatment

**Figure 1:**
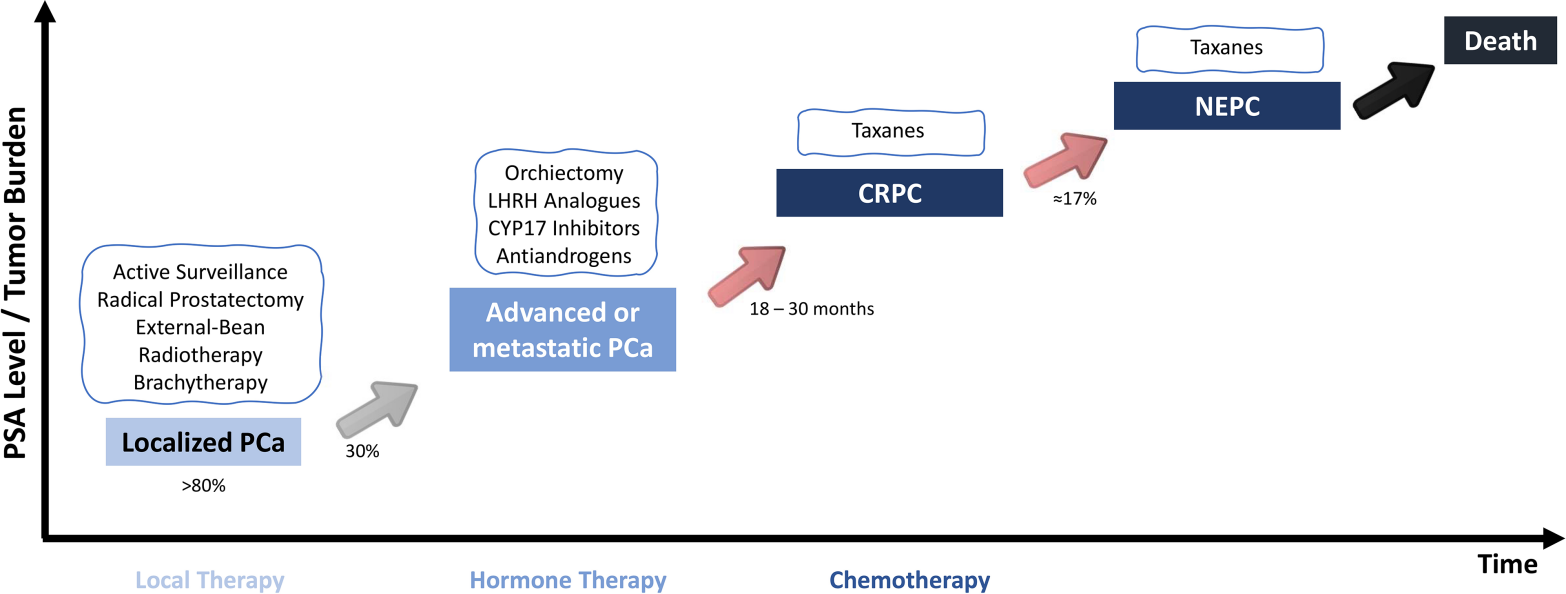
Standard of care for the different PCa stages. For Localized PCa, RP is the interventional standard of care. On the other hand, for advanced and metastatic disease, ADT is the recommended treatment. However, nearly all patients stop responding ADT and progress to a CRPC, for which there are no effective treatment options. Moreover, about 17% of the patients with a castration-resistant form of the disease will develop neuroendocrine differentiation, which is independent of AR signaling pathway. PSA, prostate-specific antigen; PCa, prostate cancer; CRPC, castration-resistant prostate cancer; NEPC, neuroendocrine prostate cancer.

Clinical parameters and tumor stage are crucial for therapy decision making in PCa, with therapeutic recommendations varying for each stage (**Figure 1**) (23, 24). For localized disease, several possibilities exist, including active surveillance and curative-intent strategies (radical prostatectomy (RP), external beam radiotherapy (EBRT) and brachytherapy) (24, 25). Additionally, for the subset of high-risk localized PCa, neoadjuvant and concurrent ADT may be considered (25). Nevertheless, in approximately 30% of cases that undergo curative-intent treatment, disease progression develops, accompanied with lymph node invasion and/or metastatic dissemination. For these patients, ADT with luteinizing hormone-releasing hormone (LHRH) agonists, anti-androgens, or surgical castration is recommended (26–29). Initially, ADT typically leads to 90-95% decrease in circulating androgen levels, being complemented by a 50% decrease in intraprostatic dihydrotestosterone (DHT) and AR inhibition (30), impairing tumor cells’ survival (26, 28). However, within 18-30 months, cancer cells eventually become resistant to the different castration strategies (31). For CRPC, although no curative options are available, docetaxel is recommended for disease management (25). Moreover, it was reported that patients might also benefit from bicalutamide and low dose corticosteroids, which were found to control PSA levels and improve symptoms, although no increase in overall survival was depicted (32). In the beginning of 2022, the Food and Drug Administration (FDA) approved the use of the novel Novartis Pluvicto™ - Lu^177^ vipivotide tetraxetan – for the treatment of progressive, PSMA-positive metastatic CRPC (33). This novel approach, in combination with the standard of care, decreased the death risk, improved overall survival and progression-free survival of these subset of patients (33). Neuroendocrine differentiation of tumor cells is observed in 17% of CRPC patients and only palliative options are proposed for this disease state (34).

Considering PCa disease progression, herein we intent to describe the mechanisms involved in therapy resistance in PCa, highlighting new potential drug targets.

### Resistance Mechanisms

**Figure 2:**
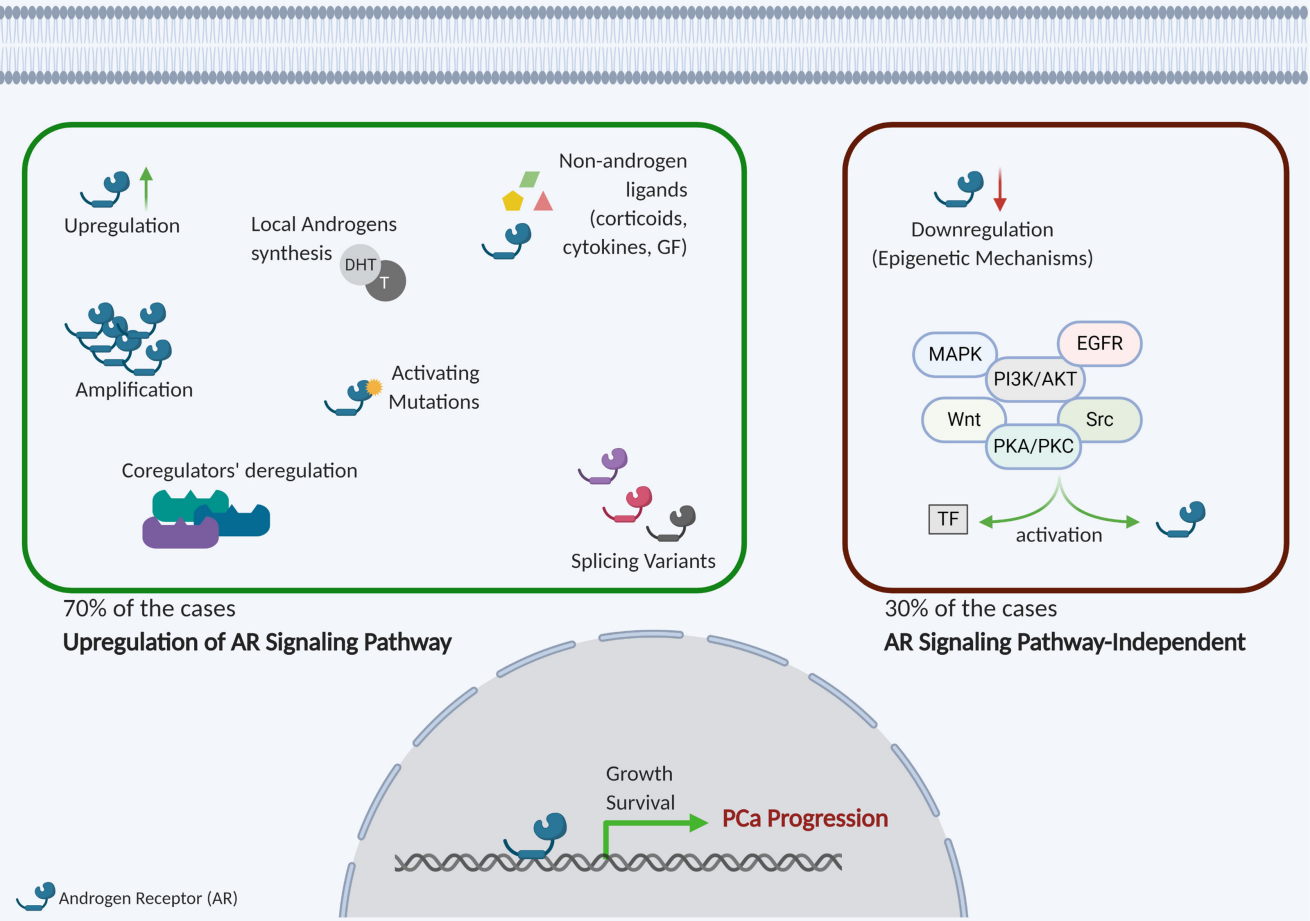
Mechanisms of resistance to androgen deprivation therapy. The process underlying ADT resistance and CRPC development involve, in 70% of the cases, upregulation of AR signaling pathway. In this case, upregulation of AR expression, amplification or activating mutations, AR splice variants, promiscuous AR activation by non-androgen ligands and deregulation of AR coactivators and co-repressors is observed. On the other hand, in 30% of the cases there is AR expression downregulation and activation of other signaling pathways involved in cell survival and growth activation. AR, androgen receptor; T, testosterone; DHT, dihydrotestosterone; GF, growth factors: TF, transcript factors. Created with BioRender.com.

During treatment of advanced and metastatic PCa, most patients develop resistance to ADT (31, 35) and although this process is not fully understood, several mechanisms were reported to be involved in the acquisition of the castration-resistant state (**Figure 2**). Regardless of castrate levels of testosterone, tumor cells can proliferate due to clonal selection of cells with AR amplification (36). Thus, an enhanced number of receptors may bind to the vestigial androgens in circulation, maintaining AR signaling (36). Moreover, gain-of-function and point mutations in *AR* results in increased activation and decreased specificity, respectively, both resulting in tumor cell survival (37–39). Decreased AR specificity allows for growth factor-induced activation (39), through insulin-like growth-factor-1 (IGF-1), keratinocyte growth factor (KGF), epidermal growth factor (EGF) (37), and fibroblast growth factor (FGF) (40). Similarly, these growth factors also bind receptor tyrosine kinase (RTK), which can regulate AR activity (38, 40). RTK and their intracellular signaling pathways play an important role in CRPC cells’ proliferation and, among these, the ERBB family (41), PI3K (5), ERK1/2 (42), Src (43), ROR-γ (44) and the glucocorticoid receptor (GR) (45) were found hyperactivated in CRPC (41, 46). Cytokines such as TNFα, IL-6 and IL-23 have been additionally suggested to modulate AR. TNFα was shown to bind to its receptor and activate NF-kB signaling pathway (47), whereas IL-6 was involved in MAPK cascades activation (48), both triggering AR signaling. Calcinotto *et al.* further reported that IL-23, secreted by myeloid-derived suppressor cells (MDSCs), activates the STAT3-RORγ pathway, by binding to IL-23R on tumor cell surface, culminating in AR activation (49). Subsequently, AR binds to androgen-responsive elements (ARE) on DNA, and in association with different co-regulators, promotes gene expression (50). Importantly, when binding occurs in DNA repair genes’ regulatory regions, especially of *PARP1*, *Ku-70*, *Ku-80* (51) and *TOP2B* (52), genomic rearrangements and DNA double stranded breaks may occur (53). The well-known *TMPRSS2:ERG* fusion can interact with the DNA repair protein and AR co-regulator PARP1, mediating transcription, invasion, and metastization (54). In AR-positive cells, GATA2, under the NOTCH family regulation, acts as an AR co-activator, maintaining AR signaling (55). Furthermore, different AR variants derived from alternative splicing have been shown to be involved in the acquisition of androgen-independent state (56). In CRPC, the most well described is the constitutively active AR-V7, which lacks the ligand-binding domain (LBD) and has an effective role in activating transcription (57). Epigenetic aberrations also contribute to post-ADT progression. In 30% of CRPC cases, *AR* expression might be completely lost and hypermethylation and histone post-translational modifications seem to be implicated in this process (2, 58, 59).

After resistance to first-line ADT, second generation anti-androgens (e.g., enzalutamide, abiraterone acetate) were found to improve survival of CRPC patients. Nonetheless, tumor cells eventually become resistant due to AR signaling reactivation (60). A specific kinase, AURKA, which is involved in chromosome instability, was found overexpressed in AR-positive CRPC cells (60). Kivinummi and colleagues showed that AURKA expression was directly targeted by androgens, with the AR specifically binding to the gene regulatory regions, resulting in reduced progression-free survival (60).

For patients harboring CRPC, taxane-based chemotherapy is the only therapeutic option which increases survival. However, patients eventually become resistant to docetaxel treatment (61). Cancer cells expressing *Mdr1* might be selected after therapy pressure, leading to decreased docetaxel intracellular intake (62). Moreover, alterations in microtubule-associated proteins’ expression result in decreased docetaxel efficacy (63). Indeed, tubulin isoform βIII overexpression correlated with docetaxel resistance in CRPC (63, 64).

Although recently approved (33), approximately 1/3 of the PSMA-positive CRPC patients do not benefit from the Lu^177^ vipivotide tetraxetan PSMA-based targeting (65, 66). Several studies have already pinpointed the PSMA heterogenic expression, defect on DNA repair genes, clonal expansion of PSMA-negative cells and tumor heterogeneity as possible mechanism of resistance (66). A particular work reported, in a mouse model, that TP53-negative tumors were less responsive to treatment, compared to TP53 wild-type tumor-bearing mice, highlighting a potential resistance mechanism (65) and a need for assessing resistance in further studies.

Furthermore, tumor microenvironment (TME) has been shown to be an important driver of resistance to ADT and taxane-based chemotherapy. The stromal component might promote CRPC progression through vascularization, apoptosis inhibition and epithelial mesenchymal transition (EMT) promotion (67). Specifically, cancer-associated fibroblasts (CAFs) are known to stimulate mesenchymal phenotype through αSMA (68), and besides promoting cancer progression through EMT-related mechanisms, TGFβ-dependent activation leads to growth factor secretion and sustainment of cancer cells survival (69).

## Methods

A PubMed search was carried out, using the query (AR mutations OR AR variants OR γ-secretase inhibitor OR HERB inhibitor OR PI3K inhibitor OR AKT inhibitor OR mTOR inhibitor OR glucocorticoid receptor inhibitor OR ROR inhibitor OR IGFR inhibitor OR MAPK inhibitor OR AUKRA inhibitor OR Scr inhibitor OR MET inhibitor OR STAT3 inhibitor OR IL-23 antibody OR TOP2 inhibitor OR BET inhibitor OR HAT inhibitor OR HDAC inhibitor OR HMT inhibitor OR HDM inhibitor OR DNMT inhibitor) AND (prostate cancer), with the time interval from 2010 to 2022. Additionally, 23 research articles prior to 2010 covering relevant data were included. Only original research articles, written in English, and those including *in vitro* and/or *in vivo* pre-clinical studies reporting drug screening assays in prostate cancer were considered. The records were imported to the reference manager EndNote. Subsequently, all abstracts were critically evaluated and only those providing relevant information for the present topic were selected. Our aim was to address the recently reported targeted therapies and potential combinations that may improve disease management and care in PCa patients.

**Figure 3:**
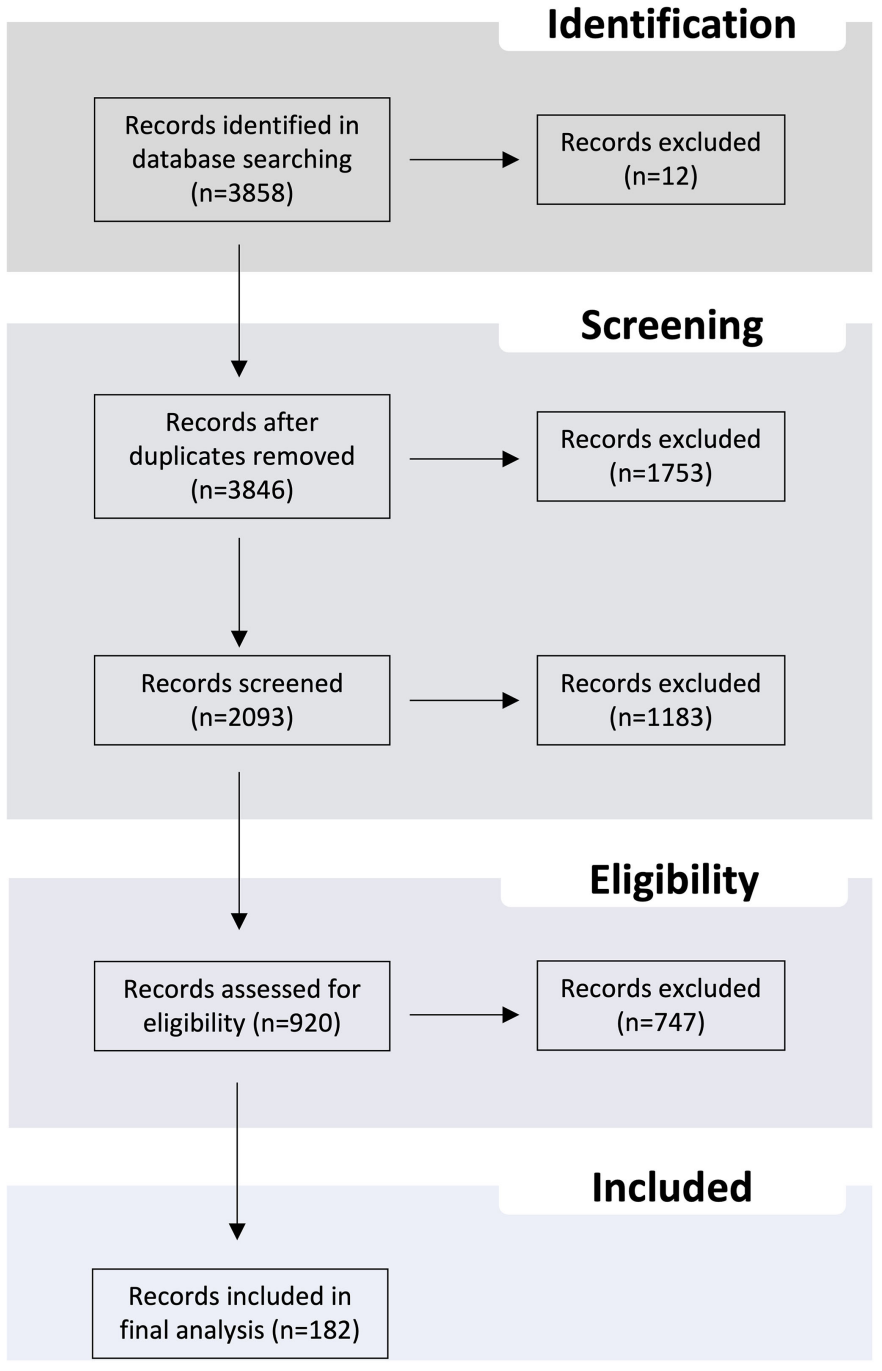
Flux gram presenting the summary of the methodology used in this review.

A summary of the methodology is provided in **Figure 3**.

## Targeted Therapies

Having in mind that the aforementioned molecular alterations may account for PCa therapy resistance, we focused on the development and pre-clinical screening of new and effective targeted therapies enabling Precision Medicine. Hence, we aimed to emphasize the current state of targeted therapies’ screening in PCa, unveiling their potential clinical use.

### Potential Targets for PCa Management

Because AR-dependent mechanisms are associated with 70% of ADT-resistant PCa cases (2), targeting the AR itself, its splicing variants or the associated co-regulators might have substantial therapeutic impact in CRPC. In the past few years, drug targeting of AR mutants, variants, and co-regulators has been shown to have anti-tumoral effects in AR-positive CRPC cells (**Table 1**). Galeterone, a CYP17A1 inhibitor, causes AR T878A mutant degradation and blocks transcription of AR target genes (70), whereas niclosamide induces AR-V7 protein degradation (75). This new AR target approach is under evaluation in clinical trials enrolling PCa patients (**Supplementary Table 1**). Nevertheless, for most of the described drugs, the anti-neoplastic effect was based on AR N-terminal blocking or AR splicing inhibition, ultimately impairing AR-driven PCa cell proliferation (**Table 1** and **Supplementary Table 1**). Additionally, indirect AR inhibition might be achieved by diminishing the activity of the positive co-regulators of the receptor transcription activity, such as GATA2 and ONECUT2 (**Table 1**), whose inhibition was reported to not only reduce cell proliferation (**Supplementary Table 1**), but also synergize with ADT agents (80, 81, 83) and docetaxel (82), displaying enhanced efficacy.

**Table 1:** Potential targets and drugs for the management of therapy resistant prostate cancer.

Target	Drug	Mechanism of action	Combination	References
AR mutations	Galeterone	AR T878A mutant degradation	N.a.	(70)
AR variants	EPI-506	Inhibits AR N-terminal domain	BEZ235 (PI3K/Akt inhibitor)	(71, 72)
	EPI-001		N.a.	(73, 74)
	Niclosamide	AR-V7 degradation	N.a.	(75)
	Thailanstatins	Inhibits AR splicing	N.a.	(76, 77)
	Peptidomimetic D2	Targets the transactive domain of AR-V	N.a.	(78)
	ONC201/TIC10	Targets AR-fl and AR-V7	Enzalutamide, docetaxel, everolimus (mTOR inhibitor)	(79)
Co-regulators	RO4929097	inhibits y-secretase, impairing AR co-activator GATA2 activity	Abiraterone	(75, 80)
	PF-3084014/PF-03084014/nirogacestat		Standard ADT, docetaxel	(80–82)
	DAPT/GSI-IX		Abiraterone	(80, 83, 84)
	BMS-708163/avagacestat		Enzalutamide	(80)
	CSRM617	inhibits ONECUT2 function	N.a.	(85)
Bypass Signaling	PKI 166	HerB1 and ErbB2 inhibitor	STI571 (PDGFR inhibitor), paclitaxel	(86–88)
	ZD1839/gefitinib		Enzalutamide, paclitaxel, ERK1/2 and PI3K inhibitors	(89–96)
	3BrQuin-SAHA & 3ClQuin-SAHA	EGFR inhibitor	N.a.	(97)
	Spautin-1		Standard ADT	(98)
	ZINC05463076 or ZINC2102846 or ZINC19901103		N.a.	(99)
	PD168393		N.a.	(100)
	CUDC-907	PI3K inhibitor	N.a.	(101)
	BAY1082439		N.a.	(102, 103)
	SF2523		N.a.	(104)
	LASSBio-2208		N.a.	(105)
	ZSTK474		N.a.	(106, 107)
	isorhamnetin		N.a.	(108)
	4-Acetylantroquinonol B		N.a.	(109)
	BEZ235/dactolisib	Dual PI3K and mTOR inhibitor	Docetaxel	(110–113)
	GDC-0068/Ipatasertib	AKT inhibitor	Enzalutamide	(114–116)
	MK-2206		N.a.	(117, 118)
	AZD5363		Standard ADT	(119–122)
	GNE-493		N.a.	(123)
	RAD001/everolimus	mTOR inhibitor	Docetaxel	(124–127)
	MK-2206	AKT and mTOR dual inhibitor	MK-8669	(128, 129)
	CB-03-10	Glucocorticoid receptor inhibitor	N.a.	(130)
	RU486/mifepristone		Docetaxel	(131–133)
	XY018	RORγ inhibitor	N.a.	(134)
	GSK805		N.a.	(134)
	SR2211		N.a.	(134)
	MP470/amuvatinib	RTK inhibitor	Erlotinib (EGFR inhibitor)	(135)
	GSK1838705A	IGFR1 inhibitor	N.a.	(136)
	NVP-AEW541		N.a.	(137)
	AZ12253801		N.a.	(138)
	PD325901/mirdametinib	MAPK/ERK inhibitor	N.a.	(139)
	U0126	MEK/ERK inhibitor	N.a.	(140)
	MLN8237/alisertib	AUKRA inhibitor	N.a.	(60)
	BMS-354825/dasatinib	Src inhibitor	BMS-754807 (IGF1 inhibitor)	(141–147)
	AZD0530/saracatinib		N.a.	(148, 149)
	SKI-606/bosutinib		N.a.	(150)
	BMS-777607	c-MET inhibitor	N.a.	(151, 152)
	GPB730	STAT3 inhibitor	Anti-CTLA-4	(153)
	Acacetin		N.a.	(154)
	GAP500/galiellalactone		Standard ADT	(155–157)
	EC-70124		N.a.	(158)
Cytokines	G23-8	Antibody against IL-23	Enzalutamide	(49)
DNA repair pathway	AZD-2281/olaparib	PARP inhibitor	N.a.	(159–162)
	ABT-888/veliparib		N.a.	(163, 164)
	AZD2461		N.a.	(165, 166)
	Rucaparib		N.a.	(167)
	VP-16/etoposide phosphate	TOP2 inhibitor	N.a.	(168)

N.a., not applicable; ADT, androgen deprivation therapy; AR-fl, androgen receptor full length; AR-V, androgen receptor variant.

Conversely, 30% of the advanced and metastatic PCa cases progress due to AR bypass mechanisms, which allow tumor cells to survive in an AR-independent manner (2). As previously described, a significant proportion of the bypass is based on RTK intracellular signaling activation, that constitutes a putative therapy target in resistant PCa (2). Many of the existent pre-clinical studies target the HerbB family, PI3K, mTOR, Akt, GR, RORγ, IGFR, MAPK, Src and STAT3 (**Table 1** and **Supplementary Table 1**). Generally, drug treatment inhibited the specific target activity, reduced tumor cell proliferation and viability, and promoted apoptosis (**Supplementary Table 1**), displaying anti-tumoral effects both *in vitro* and *in vivo*.

Nonetheless, as reported for the drugs that target AR co-regulators, the most promising results were achieved when combining a targeted therapy with the standard therapy strategies. For example, the Akt inhibitor ipatasertib, under test in clinical studies in PCa (**Supplementary Table 1**), re-sensitized CRPC cells to antiandrogens, when combined with enzalutamide, inducing apoptosis, and leading to remarkable tumor cell growth inhibition, both *in vitro* and *in vivo* (114). Gefitinib (89), BEZ235 (110), RAD001 (124) or RU486 (131) were also reported to re-sensitize resistant cells to standard chemotherapy agents (**Supplementary Table 1**). Interestingly, CUDC-907 (101), CB-03-10 (130) and MP470 (135), in addition to selective RTK inhibition, were found to inhibit HDAC6, AR and EGFR, respectively. These drugs caused cytotoxic effects in resistant cells (**Supplementary Table 1**), indicating a possible benefit of targeting multiple pathways for management of resistant PCa.

Although most of the drugs listed in **Table 1** have demonstrated good therapeutic potential, in some cases a possible resistant mechanism was also identified. The PI3K inhibitor CUDC-907 resulted in increased phospo-ERK levels (101), whereas PD325901, by inhibiting the ERK pathway, induced hyperactivation of the pro-proliferative PI3K and hedgehog pathways (139). In both studies, compensatory signaling mechanisms were suggested as the cause of resistance, thus, reinforcing the benefit of combinatory strategies to enhance anti-tumoral effects.

Furthermore, the combination of standard radiation therapy and PARP inhibitors was shown to have a significant effect on tumor cells viability (**Table 1** and **Supplementary Table 1**). Specifically, veliparib (163) and rucaparib (167), two drugs under clinical investigation in PCa (**Supplementary Table 1**), were shown to re-sensitize CRPC cells to radiotherapy, impairing tumor cell growth. Moreover, this class of inhibitors similarly synergized with ADT agents (159, 160), AUKRA inhibitors (161) and epi-drugs (164, 165), with improved anti-neoplastic effects.

### Targeting Epigenetics for PCa Treatment

Epigenetic alterations have been recognized as a hallmark of cancer (169), and since it comprises reversible modifications (59), there is a potential for drug targeting. FDA has approved two drugs that target epigenetic players, 5-azacytidine and 5-aza-2′-deoxycytidine, for myelodysplastic syndrome treatment (170). These two drugs, as DNA methyltransferases (DNMTs) inhibitors, are incorporated into DNA, inhibiting DNMT activity and decreasing global methylation levels (171, 172). Nevertheless, there is a potential for targeting the entire epigenetic machinery in cancer treatment, as we have previously reported (173). In therapy resistant PCa, histone acetyltransferases (HATs), HDACs, histone demethylases (HDMs), histone methyltransferases (HMTs), BET, and DNMTs inhibitors are currently under pre-clinical and clinical studies (**Table 2** and **Supplementary Table 2**), displaying anti-tumoral effects, mostly due to enzyme inhibition and gene expression reprograming (**Supplementary Table 2**). Interestingly, the most promising results were found when epi-drugs were combined with standard ADT compounds (188, 209, 224, 228, 229, 241, 243), docetaxel (175, 197, 210, 217, 234), radiation therapy (203) or other epi-drugs (196, 204, 235), suggesting an interplay between epigenetic and non-epigenetic targeting in PCa management.

**Table 2:** Potential epigenetic targets and epi-drugs for the management of therapy resistant prostate cancer.

Target	Drug	Mechanism of action	Combination	References
BET	OTX015/MK-8628/birabresib	BRD2/3/4 inhibitor	N.a.	(174)
	JQ1	BRD4 inhibitor	N.a.	(175–182)
	GSK1210151A		N.a.	(180)
	Y08060		N.a.	(183)
	CPI-203		N.a.	(184)
	AZD5153		N.a.	(185)
	I-BET151		N.a.	(180)
	SF2523		N.a.	(104)
	WWL0245		N.a.	(186)
	I-BET762/molibresib	BET inhibitor	N.a.	(187)
	ZEN-3694		Enzalutamide	(188, 189)
	ABBV-744		N.a.	(190)
	Y06014		N.a.	(191)
	NEO2734		N.a.	(192)
	dBET6	BET protein degradation	N.a.	(175)
HAT	A-485	CBP/p300 inhibitor	N.a.	(193)
	CCS1477		N.a.	(194)
	Y08197		N.a.	(195)
	I-CBP112		A-485	(196)
HDAC	Trichostatin A/TSA	HDAC I and II inhibitor	Bortezomib (proteosome inhibitor), chemotherapy	(197–202)
	Panobinostat/LBH-589	Pan HDAC inhibitor	Hydralazine (DNMT inhibitor), RT, zoleronic acid	(203–208)
	Vorinostat/SAHA	HDAC I inhibitor	Bicalutamide, docetaxel	(209–212)
	MHY219		N.a.	(213, 214)
	Jazz90 & Jazz167	HDAC inhibitor	N.a.	(215, 216)
	CG200745		Docetaxel	(217)
	MHY4381		N.a.	(218)
	Valproic Acid/VPA		N.a.	(5, 17, 219, 220)
	A248		N.a.	(221)
	MPT0B451		N.a.	(222)
	2-75	HDAC 6 inhibitor	N.a.	(223)
HMT	GSK-343	EZH2 inhibitor	Standard ADT, metformin	(224)
	Tazemetostat/EPZ-6438		N.a.	(225)
	GSK-926		N.a.	(226)
	LG1980		N.a.	(227)
	GSK-126		N.a.	(228–231)
HDM	NCL1	LSD1 inhibitor	Docetaxel	(232, 233)
	HCI-2509		N.a.	(234)
DNMT	5-AZA-2’-deoxycytidine/decitabine	DNMT inhibitor	Sodium butyrate	(171, 235–240)
	5-azacytidine/azacytidine		Standard ADT	(172, 241)
	RG108		N.a.	(242)
	Hydralazine		Panabinostat	(204)

N.a., not applicable; ADT, androgen deprivation therapy; BET, Bromodomain and Extra-Terminal motif; HAT, histone acetyltransferase; HDAC, histone deacetylase; HMT, histone methyltransferase; HDM, histone demethylase; DNMT, DNA methyltransferase; RT, radiation therapy.

Remarkably, BET inhibitors such as JQ1, GSK1210151A and I-BET151 were found to decrease AR-fl and AR-V7 expression and activity (**Supplementary Table 2**), demonstrating a potential to be used with both an epigenetic- and AR-targeting purpose. However, disadvantageous off-target effects were observed after JQ1 treatment. JQ1 was found to promote PCa cell invasion and metastatic potential due to FOXA1 inactivation in a BET-independent manner (176). Therefore, high *FOXA1* expression tumors are not suitable for JQ1 treatment (176), highlighting the importance of personalized strategies, based on tumor cell biology, for PCa management.

Notwithstanding all the work that has been accomplished in the epi-drug field, the role of different epigenetic enzymes in cancer, particularly PCa, and its potential targeting for a reprograming purpose remains largely unknow. Therefore, an investment in this field of research might contribute to improve the management of, not only therapy resistant PCa, but also other cancers displaying therapeutically relevant epigenetic modifications.

### Immunotherapy-Based Therapies for PCa Management

PCa has long been described as a *“cold”* tumor, characterized by an immune-suppressive environment (244). However, in the last decade, several efforts have been made to overcome this feature. This includes the use of different immune therapies, alone or in combination with the standard of care (**Table 3** and **Supplementary Table 3**). Published data includes reports from clinical trials targeting PD-L1, PD-1, CTLA-4, and the approved cellular immunotherapy Sipuleucel-T (**Table 3**). Overall, immunotherapy did not significantly improve the survival of PCa patients, but the effect on PSA was promising (**Supplementary Table 3**). Interestingly, the most encouraging results were obtained by the combination of pembrolizumab (anti-PD-1 drug), with the ADT enzalutamide (246). These results highlight the need for pre-clinical *in vitro* studies aiming at understanding the molecular mechanisms behind the *“cold”* PCa microenvironment, paving the way to further studies of novel immune-based therapies.

**Table 3:** Immunotherapy for the management of therapeutic resistant PCa.

Target	Drug	Mechanism of action	Combination	References
PD-L1	Atezolizumab	Inhibits PD-L1	Enzalutamide	(245)
PD-1	Pembrolizumab	Targets PD-1	Enzalutamide, docetaxel, prednisone	(246–251)
Cellular Therapy	Sipuleucel-T	Cellular immunotherapy	N.a.	(252)
CTLA-4	Ipilimumab	Inhibits CTLA-4	ADT	(253–256)
	Tremelimumab		Bicalutamide	(257)

N.a., not applicable; ADT, androgen deprivation therapy.

## Conclusion

Herein, we described the mechanisms underlying the acquisition of therapy resistance in PCa, and potential targetable molecules, listing druggable targets in resistant disease and addressing pre-clinical studies describing the anti-tumoral effects of several drugs. We provided insight on innovative PCa treatments, to be exploited in pre-clinical studies and, if successful, in clinical trials, allowing for improved treatment of CRPC patients. Although several targeted therapies are already under clinical trials in PCa, there is a need for a more personalized analysis of tumor cell biology, enabling the selection of the most suitable therapeutic strategy, improving the management of resistant disease.

## Author Contributions

Conceptualization, FM-S and CJ. Systematic review of literature, FM-S. Writing—original draft preparation, FM-S. Writing—review and editing, RH and CJ. Image editing, FM-S. Supervision, CJ. All authors have read and agreed to the published version of the manuscript.

## Funding

CJ research is supported by the Research Center of Portuguese Oncology Institute of Porto (CI-IPOP-27-2016). FM-S contract was funded by Porto Comprehensive Cancer Center (Porto.CCC, Contract RNCCCP.CCC-CI-IPOP-LAB3-NORTE-01-0145-FEDER-072678).

## Conflict of Interest

The authors declare that the research was conducted in the absence of any commercial or financial relationships that could be construed as a potential conflict of interest.

## Publisher’s Note

All claims expressed in this article are solely those of the authors and do not necessarily represent those of their affiliated organizations, or those of the publisher, the editors and the reviewers. Any product that may be evaluated in this article, or claim that may be made by its manufacturer, is not guaranteed or endorsed by the publisher.

## Abbreviations

AR, androgen receptor; AR-fl, androgen receptor full length; AR-V, androgen receptor variant; ADT, androgen-deprivation therapy; ARE, androgen-responsive elements; BET, bromodomain and extra-terminal motif; CAFs, cancer-associated fibroblasts; CRPC, castration resistant prostate cancer; DHT, dihydrotestosterone; DNMTs, DNA methyltransferases; EGF, epidermal growth factor; EMT, epithelial mesenchymal transition; EBRT, external beam radiotherapy; FGF, fibroblast growth factor; FDA, Food and Drug Administration; GR, glucocorticoid receptor; GF, growth factors; HATs, histone acetyltransferases; HDACs, histone deacetylases; HDMs, histone demethylases; HMTs, histone methyltransferases; IGF-1, insulin-like growth-factor-1; KGF, keratinocyte growth factor; LBD, ligand-binding domain; LHRH, luteinizing hormone-releasing hormone; MDSCs, myeloid-derived suppressor cells; NEPC, neuroendocrine prostate cancer; N.a., not applicable; PCa, Prostate cancer; PSA, prostate-specific antigen; RT, radiation therapy; RP, radical prostatectomy; RTK, receptor tyrosine kinase; T, testosterone; TF, transcript factors; TME, tumor microenvironment.
