# Supplementary figures and images for: From Therapy Resistance to Targeted Therapies in Prostate Cancer
Source: Front Oncol. 2022 May 24;12:877379. doi: 10.3389/fonc.2022.877379 (PMC9170957; doi:10.3389/fonc.2022.877379)

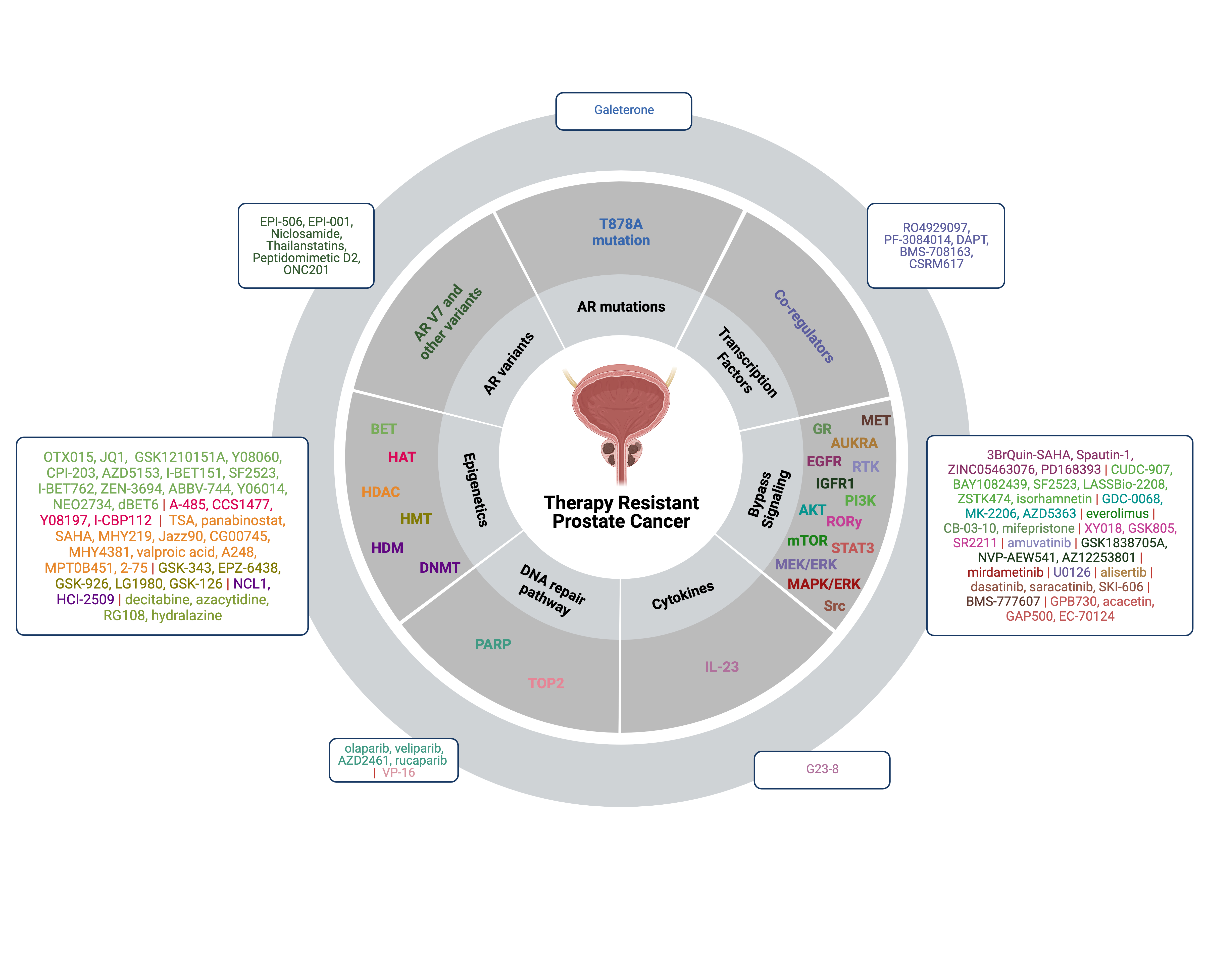

Supplement: Supplementary file 1 [file Image_1.jpeg]
